# Antibiotic Resistance and Its Impact on Disease Management

**DOI:** 10.7759/cureus.38251

**Published:** 2023-04-28

**Authors:** Chinmayee A Nadgir, Dalia A Biswas

**Affiliations:** 1 Department of Physiology, Jawaharlal Nehru Medical College, Datta Meghe Institute of Medical Sciences, Wardha, IND

**Keywords:** antibiotic resistance genes, antibiotic resistance programs, tuberculosis, inappropriate use of antibiotics, antibiotic resistance

## Abstract

Antibiotic resistance has been a challenge to the medical fraternity and has had a massive impact on disease management. The overuse of antibiotics and careless prescription by doctors have been one of the primary reasons for the development of antibiotic resistance among the masses. This article draws attention to the significant reasons causing antibiotic resistance, such as overuse, antibiotic resistance genes, and extensive use of antibiotics in agriculture. It also brings forward the challenges posed by antibiotic resistance in the management of various diseases like tuberculosis, COVID-19, and vancomycin-resistant enterococci infections. The article includes a case study that depicts the threat posed by antibiotic resistance in tuberculosis treatment. This article also shows the effects of antibiotic resistance on COVID-19 patient care and treatment.

It further includes methods that can be implemented on international levels as well as individual ground levels to curb antibiotic resistance. One of the methods has a recent finding in which proteins produced in the body are being modified and used in treatments to reduce the use of antibiotics, which ultimately serves the goal of curbing antibiotic resistance by reducing overuse.

## Introduction and background

Antibiotics are one of the wonder discoveries of the twentieth century [1]. Antibiotic resistance has been a "hidden" epidemic. In India, antibiotic resistance is single-handedly killing more people than cancer and road traffic accidents combined deaths killing 700,000 people every year, and another 10 million are anticipated to die from it by the year 2050 [2]. Medical experts have warned us that the pre-antibiotic period is returning; a current database lists the existence of more than 20,000 possible resistance genes expected to exist from accessible bacterial genome sequences of roughly 400 different types [1]. India has been referred to as the antibiotic-resistant capital of the world. Antibiotic resistance claims the lives of about 23,000 patients each year in the United States and results in an increase in healthcare costs of over $20 billion [3]. Antibiotic resistance is a multifaceted public health issue. As the efficacy of the antibiotics used to treat them declines, an increasing range of illnesses, including salmonellosis, tuberculosis, gonorrhea, and pneumonia, are becoming more exigent to treat. Acute respiratory diseases were deemed as the most inappropriate antibiotic use in 2015, accounting for 50% of all outpatient antibiotic prescriptions [4]. The ultimate, well-known results of such cumulative evolutionary events are ever greater challenges in preventing and treating bacterial illnesses [4]. Meningitis and pneumonia are two illnesses that are more challenging to treat. The interconnected domains of One Health are influenced by the formation, evolution, and transfer of antibiotic-resistant bacteria on a local and global scale [5]. You might require more potent, costly medications. Primarily focusing on tuberculosis, although tuberculosis is an infectious disease that may be treated, an estimated 1.5 million people died from it in 2020, and one of the main causes of this is that the disease is continuing to develop drug resistance [6]. Only about one in three people with multidrug-resistant tuberculosis (MDR-TB) were able to access treatment in 2020. One of the most pressing and difficult concerns in tuberculosis control today is the ongoing spread of MDR-TB [6]. The incorrect use of antibiotics in one patient could result in the emergence of a resistant strain that spreads to people who do not take antibiotics, and this poses a serious public health risk. Although India was the hub of tuberculosis, it was on its way to eliminating tuberculosis by 2025. However, after the beginning of COVID-19, antibiotic resistance posed a significant hurdle in eliminating tuberculosis.

## Review

Methodology

To search the Central database and MEDLINE, we used Cochrane Library and PubMed. For each database, the following search approach was created: ((Antibiotic resistance) AND ("Tuberculosis drugs") AND ("Antibiotic Resistance Genes")) : ("effects on viral infection treatment") AND ("methods to reduce antibiotic resistance. In addition, we looked over the relevant papers' references list. Studies that were found through these computerized searches. as well as pertinent sources included in their bibliographies, were examined. In the end, this review article featured a total of 35 articles.

Causes of antibiotic resistance

Bacteria can respond to a wide array of environmental threats like extreme heat, cold, and even alkalinity and salinity; furthermore, they can even react to the presence of antibiotic molecules that can jeopardize their existence due to remarkable genetic plasticity [7]. Antibiotic resistance is mainly caused due to three reasons: antibiotic resistance genes, inappropriate use and prescription of antibiotics, and extensive use in agriculture. Humans are mostly exposed to resistant diseases through other individuals, whether in hospitals or in communal settings. Humans are mainly exposed to resistant pathogens from other people, either in hospitals or community settings. Common dispersal routes are via body contact or indirect contact by aerosols and food [8].

Antibiotic-Resistant Genes

Many human gene transfer (HGT) methods free genes from typical vertical inheritance. By concentrating antibiotic-resistant genes (ARGs) in a single cell, HGT promotes the emergence of incurable "superbugs." One of the most known examples is *Staphylococcus* resistance to penicillin, indicated by an enzyme (penicillinase) that destroyed the drug [9]. A genetic material jumps between strains and species due to mechanisms like conjugation by plasmids, transduction by bacteriophages, and natural transformation by extracellular DNA. Active efflux, reduced permeability, ribosome modification, and inactivation of medicines by aminoglycoside-modifying enzymes are a few of the resistance mechanisms that have been discovered.

Inappropriate Use and Prescription of Antibiotics

According to estimates, 30% of the annual millions of prescriptions supplied are unnecessary. Over the past few decades, *S. aureus* has increasingly developed a higher level of drug resistance as a result of bacterial evolution and antibiotic misuse. Methicillin-resistant *Staphylococcus aureus* (MRSA) infection rates have increased globally, and its clinical anti-infective therapy has been increasingly challenging. Research has shown that the resistance mechanisms of S. aureus are exceedingly intricate, particularly for MRSA, which is resistant to a wide range of antibiotics [10]. Investigations were carried out on children's gut resistance four years after receiving twice-yearly azithromycin distributions. All kids aged 1 to 59 months received a mass distribution of either azithromycin or a placebo every six months for four years. The determinants of macrolide resistance were 7.4 times greater (95% CI, 4.0 to 16.7) at 36 months and 7.5 times higher (95% CI, 3.8 to 23.1) at 48 months in the azithromycin group compared to the placebo group. Ongoing mass distributions of azithromycin are also scrutinized for indicators of non-macrolide resistance, such as resistance to beta-lactam antibiotics, which are commonly prescribed in this part of Africa [11]. Since their discovery, lactams (penicillins) have been used to treat severe bacterial infections in humans. Their mechanism of action is that these antibiotics bind to penicillin-binding proteins and prevent Gram- and Gram-negative bacteria from synthesizing peptidoglycan or cell walls [12]. The introduction of new antibiotics has led to the emergence of increased antibiotic resistance.

The Extensive Use of Antibiotics in Agriculture

Prescriptions for antibiotics are part of the therapeutic regimens used to eradicate infections in humans. Antibiotic use is not only restricted to clinical settings [8,13]. The environmental microbiome is also affected by the agricultural use of antibiotics. Ninety percent of the antibiotics administered to animals are expelled in urine and faces, where they are then dispersed through fertilizers, groundwaters, and surface runoff, which are some of the pathways for environmental microbiome deterioration. Moreover, fruit trees are sprayed with herbicides like streptomycin and tetracyclines. Despite the fact that this use of antibiotics makes up a significantly lower portion of overall antibiotic use, the resulting regional dispersion can be significant. As a result of being exposed to growth-inhibiting substances, environmental microorganisms become more resistant compared to susceptible microorganisms, changing the ecology of the environment [14]. In both children and adults, several cleaning and hygienic agents that are antibacterial limit the development of immunity to antigens present in the environment. This compromises immune system versatility, which could increase morbidity and mortality due to infections that otherwise would not be intensely noxious.

Consequences of antibiotic resistance

Various diseases and their hindered management because of antibiotic resistance are discussed below.

Antibiotic Resistance in Tuberculosis

*Mycobacterium tuberculosis* populations that may momentarily withstand antibiotic pressure in the absence of changes that give drug resistance can limit effective tuberculosis treatment [15]. In a study conducted across nine countries overall, 14.8% of patients with MDR tuberculosis starting treatment with second-line drugs acquired resistance to fluoroquinolones, a second-line injectable, or both during treatment [16]. Drug resistance develops when anti-tuberculosis medications are abused due to improper prescription by healthcare professionals, usage of subpar medications, and early treatment discontinuation by individuals [6]. In several nations, including India, extensively drug-resistant tuberculosis (XDR-TB) has emerged as a fresh challenge to the control of tuberculosis [17]. A case of coinfection of chronic hepatitis B and an MDR-TB patient presenting COVID-19 shows that in 2020, a 34-year-old male patient presented to the hospital with symptoms of COVID-19 and a history of hepatitis B and MDR-TB [18]. On assay of sputum, *Mycobacterium tuberculosis* resistant to rifampicin was found [19]. He did not respond to the first line of treatment due to this, which caused further complications. Within a week of starting bedaquiline, the patient showed considerable improvement and was weaned off oxygen [15].

Antibiotic Resistance in COVID-19

More than 29,400 people died in the first year of the epidemic from illnesses that are frequently linked to healthcare [20]. The COVID-19 pandemic has further contributed to increased antibiotic resistance as a large proportion of patients infected with SARS-CoV-2 were initially treated with antibiotics [20]. In a research conducted by a Chinese adult infectious disease center, antibiotics were given to 71% of COVID-19 patients who were hospitalized [21]. As per data analysis, it has been observed that children who were given antibiotics for COVID-19 treatment were associated with an increased risk of fatal outcomes. This is one of the most hazardous consequences of antibiotic resistance in patients with COVID-19 [22].

Other Life-Threatening Bacterial Infections That Have Antibiotic Resistance

The female vaginal system and the human digestive tract are frequently colonized by enterococci bacteria. Hospitals and healthcare facilities are major hubs for vancomycin-resistant enterococci infections. Patients with underlying health conditions are more vulnerable to infection by these bacteria. Antibiotic resistance in *H. pylori* has alarmingly increased everywhere. This has significantly impacted how well this bacterial ailment is treated [10]. An aggressive bacteria called *Pseudomonas aeruginosa* is the main cause of illness and mortality in cystic fibrosis sufferers and other immunocompromised individuals. Its remarkable capacity to resist antibiotics has made its eradication extremely difficult [23]. Since ESKAPE pathogens have genes for antibiotic resistance, there are fewer therapies available for major infections, which increases the burden of sickness and the number of people dying from unsuccessful treatments [24]. Because of the widespread use of antibiotic medications, which is largely to blame for the development of antimicrobial resistance, it has become harder to treat infections, including the DNA fragmentation index [25]. Physicians are hesitant to choose oral antibiotics because of the fast-rising *E. coli *antibiotic resistance [26]. Common ocular antibiotics including ciprofloxacin (9%) gentamicin (22%), and ceftazidime (13%) continue to have relatively low rates of resistance on average. However, there were significant differences in the rates of resistance reported in research from other nations; for instance, ciprofloxacin resistance could reach up to 33% [27]. Gram-negative infections are especially concerning since they are growing resistant to almost all available antibiotic medication alternatives, leading to circumstances similar to the pre-antibiotic era [9]. For example, in several African countries, the use of azithromycin has increased as a result of the pandemic. Hospitals often give broad-spectrum antibiotics to treat nosocomial infections, but this breeds resistance [20]. There was a significant increase in the abundance of ARG-carrying plasmids in the cohort treated with cotrimoxazole in a recent clinical trial conducted in a hospital in Cologne but not in the cohort treated with ciprofloxacin, suggesting that cotrimoxazole may contribute more effectively to the spread of resistance [28]. The WHO has alerted society that a post-antibiotic era will cause consequent frequent infections, where even small injuries may result in death if urgent action is not taken against antibiotic resistance [29].

Methods to Curb Antibiotic Resistance

**Figure 1:**
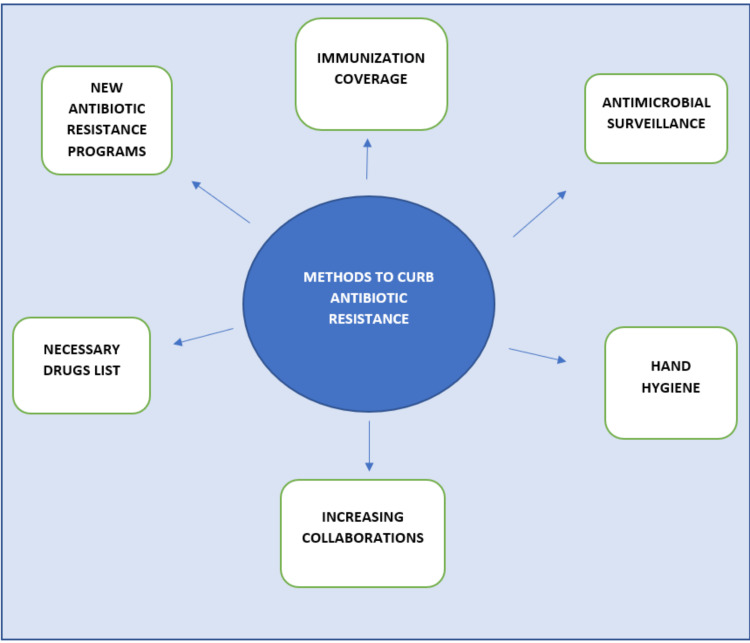
Methods to curb antibiotic resistance

The goal of One Health is to achieve the highest possible level of health for everyone, including domesticated animals, wildlife, plants, and the environment [28]. This entails implementing measures to prevent the misuse of current antimicrobials and to stop the spread of infections, as well as eliminating their inappropriate usage [30]. Although the antibiotic itself has a significant impact on antibiotic resistance, how it is utilized also plays a part in its selective role. Additionally, even though these molecules may be alien to nature to the best of our knowledge, synthetic methods like diversity-oriented synthesis, a factor of safety, and common technical documents can be employed to build unheard-of scaffolding that supports the complexity of natural products [31]. Drug resistance can be greatly impacted by the same antibiotic when administered in various ways. One study indicated that individuals' chance of carrying penicillin-resistant pneumococci increased when penicillin was administered at doses below therapeutic levels and for a duration of time (five days) [32]. Since there aren't many antibacterial targets, structurally altering vancomycin has received a lot of attention recently. Vancomycin's antibacterial potency has been increased in order to destroy strains that would otherwise be resistant. One interesting strategy for modification is to combine the properties of lipophilic membrane anchors with cationic cell-penetrating peptides as an electrostatic effector, which is anticipated to interact with the negatively charged bacterial cell wall. By combining a single lipophilic element at the same site with cationic quaternary ammonium, vancomycin's antibacterial ability can be greatly improved against resistant organisms [33]. According to a team of Australian researchers, the next weapon against antibiotic resistance is within our own immune systems. It has been found that a class of proteins called guanylate-binding proteins can identify and destroy bacteria, as well as trigger the immune system to recognize them. They could become their own treatments after a certain time period [34]. To preserve the efficiency of antibiotics, measures are mainly directed toward hospitals and drug providers that provide antibiotics without prescription [35]. The situation demands newer targets against bacterial machinery [35]. Figure 1 below describes methods to curb antibiotic resistance.

## Conclusions

Antibiotics have given the health sciences extraordinary benefits, and the rapid increase in antibiotic resistance is threatening it. This is a global crisis that reflects the worldwide overuse of antibiotics, and pharmaceutical companies are lacking to develop new antibiotics to address the challenge. Antibiotic resistance is putting the achievements of modern medicine at risk. Antibiotic-resistant infections place a substantial economic burden on the healthcare system worldwide. The world is in urgent need of coordinated efforts to curb this crisis; the international health governing bodies, along with the personal level in the community, the society, can overcome antibiotic resistance.
